# Low-frequency variability in photoplethysmographic waveform and heart rate during on-pump cardiac surgery with or without cardioplegia

**DOI:** 10.1038/s41598-020-58196-z

**Published:** 2020-02-07

**Authors:** Anton R. Kiselev, Ekaterina I. Borovkova, Vladimir A. Shvartz, Viktoriia V. Skazkina, Anatoly S. Karavaev, Mikhail D. Prokhorov, Artak Y. Ispiryan, Sergey A. Mironov, Olga L. Bockeria

**Affiliations:** 1 Department of Surgical Treatment for Interactive Pathology, Bakulev National Medical Research Center for Cardiovascular Surgery, Moscow, Russia; 2 Department of New Cardiological Informational Technologies, Research Institute of Cardiology, Saratov State Medical University, Saratov, Russia; 3 Department of Dynamic Modeling and Biomedical Engineering, Saratov State University, Saratov, Russia; 4 Laboratory of Nonlinear Dynamics Modelling, Saratov Branch of the Institute of Radio Engineering and Electronics of Russian Academy of Sciences, Saratov, Russia

**Keywords:** Cardiovascular biology, Cardiovascular biology

## Abstract

We studied the properties of low-frequency (LF) heart rate variability (HRV) and photoplethysmographic waveform variability (PPGV) and their interaction under conditions where the hemodynamic connection between them is obviously absent, as well as the LF regulation of PPGV in the absence of heart function. The parameters of HRV and finger PPGV were evaluated in 10 patients during cardiac surgery under cardiopulmonary bypass (on-pump cardiac surgery) with or without cardioplegia. The following spectral indices of PPGV and HRV were ertimated: the total spectral power (TP), the high-frequency (HF) and the LF ranges of TP in percents (HF% and LF%), and the LF/HF ratio. We assessed also the index *S* of synchronization between the LF oscillations in finger photoplethysmogram (PPG) and heart rate (HR) signals. The analysis of directional couplings was carried out using the methods of phase dynamics modeling. It is shown that the mechanisms leading to the occurrence of oscillations in the LF range of PPGV are independent of the mechanisms causing oscillations in the LF range of HRV. At the same time, the both above-mentioned LF oscillations retain their activity under conditions of artificial blood circulation and cardioplegia (the latter case applies only to LF oscillations in PPG). In artificial blood circulation, there was a coupling from the LF oscillations in PPG to those in HR, whereas the coupling in the opposite direction was absent. The coupling from the LF oscillations in PPG to those in HR has probably a neurogenic nature, whereas the opposite coupling has a hemodynamic nature (due to cardiac output).

## Introduction

Despite the relatively widespread use of photoplethysmography to assess the state of peripheral blood flow^1,2^, the question of the physiological interpretation of the frequency components of photoplethysmographic waveform variability (PPGV) remains largely debatable. Usually, the nature of high-frequency (HF) oscillations in photoplethysmogram (PPG) signal is explained by the mechanical effect of respiration^3–5^, while the low-frequency (LF) oscillations (with a characteristic frequency of about 0.1 Hz) in PPG are associated with sympathetic regulation of peripheral vascular resistance^3,6,7^. It should be noted that besides the PPG, the LF fluctuations at a similar frequency are detected also in the signals of heart rate (HR)^8,9^ and blood pressure (BP)^10,11^. Blood pressure variability (BPV) is primarily due to the vasomotor tone, which is not directly related to the heart control. Since blood flow through the distal arteries contributes to the formation of the finger PPG^12^, the autonomic regulation of BP can be indirectly assessed by the PPG signal.

In experiments with linearly increasing frequency of respiration, we have shown earlier the functional independence of LF oscillations in HR and PPG^13^. In healthy subjects, these LF oscillations are usually synchronized between themselves^14^. Quantitative estimation of the degree of synchronization between the LF oscillations in HR and PPG has potential clinical importance, for example, for assessing cardiovascular risk and controlling drug therapy^14^. However, how closely the mechanisms of the autonomic regulation of HR and BP are associated and whether they can function separately is not known. The nature of couplings between them (neurogenic or hemodynamic) also requires clarification.

The aim of this work was to study the properties and interaction of LF oscillations in HR and PPG under conditions where the hemodynamic coupling between them is certainly absent, and to study the LF regulation of PPGV in the absence of heart function. For this purpose, the parameters of heart rate variability (HRV) (if available) and finger PPGV were evaluated in patients during cardiac surgery under cardiopulmonary bypass (on-pump cardiac surgery) with or without cardioplegia. Such an approach allows us to understand better the principles of the functional organization of the autonomic regulation of blood circulation and increases the reliability of the analysis of biological signals in the experimental studies of cardiovascular system.

## Materials and Methods

### Ethical approval

The Ethics Committee of the Bakulev Scientific Center for Cardiovascular Surgery in Moscow, Russia approved the design of the study. All subjects gave informed consent. All procedures performed on humans fall under ethical regulations, including 1964 Helsinki Declaration and its later amendments.

### Patients

We studied five men during coronary artery bypass grafting, aged 60.1 ± 8.2 years (mean ± standard deviation), and five men during surgical correction of valvular heart disease, aged 57.2 ± 9.3 years.

We excluded from the study the patients with severe cardiac failures, cancer, mental illness, organic diseases of nervous system and brain, endocrine pathology except for compensated diabetes, symptomatic hypertension, or abnormalities in peripheral microcirculation.

### Surgical approach

All patients were operated using the standard cardiopulmonary bypass (CPB) (so-called, on-pump) technique. The on-pump coronary artery bypass grafting (CABG) was performed on a beating heart under the parallel perfusion and normothermia. Aorta was not cross-clamped and cardioplegic solution wasn’t administered. During the CPB a single two-stage cannula was installed into the right atrium through the free wall. For conduits we used radial artery, great saphenous vena, and the left internal thoracic artery, which in some cases was switched to internal thoracic artery from the right side. While forming the coronary anastomoses we stabilized myocardium with the “Octopus” (Medtronic, USA) or “Acrobat” (Maquet, Germany) vacuum stabilizers. After the CABG, all patients without contraindications underwent the intraoperative graft angiography in order to assess the quality of the formed anastomoses. In accordance with the standard procedure, we finished the operation with drainage of pericardial cavity and anterior mediastinum and stratification of the chest suturing wounds.

We performed the on-pump surgical correction of valvular heart disease under pharmacological cardioplegia and hypothermia. The correction included the aortic cannulation, the separate cannulation of the superior vena cava with an angled cannula, and the direct cannulation of the inferior vena cava through free wall of the right atrium. We performed the antegrade cardioplegia in aortic root or, if aortic valve was insufficient, we performed the retrograde cardioplegia through coronary sinus. While performing the cardioplegia, we administered Custodiol (Odyssey Pharmaceuticals Inc., Germany) cardioplegic solutions. In accordance with the standard procedure, we finished the operation with drainage of pericardial cavity and anterior mediastinum.

It is important to note that the flow of blood from the heart-lung machine into the aorta, distal to the place of its cannulation, does not occur laminarly, but is jerked, which is formed by the operation of a rotor motor. This generates blood flow oscillations in the peripheral channel, in particular, reflected in the PPGV. Depending on the surface area of the human body, in order to maintain the desired balance of the blood volume, the rotational speed of the rotary motor can vary in the range from 80 to 160 beats per minute. In patients without cardioplegia, the frequency of mechanical ventilation was ranged from 10 to 16 breaths per minute, while in patients with cardioplegia, this frequency was about 1–2 breaths per minute. In the case of cardioplegia, the circulating blood was cooled.

### Biological signals recording

The signals of electrocardiogram (ECG) and PPG from the middle finger of the left hand were simultaneously recorded within 10 minutes in all patients during cardiac surgery. All signals were sampled at 250 Hz and digitized at 16 bits. We used PPG sensors based on the infrared light emitting diode (wavelength of about 960 nm IR-LED) operating on reflection.

### Theoretical basis of research methods

**Figure 1:**
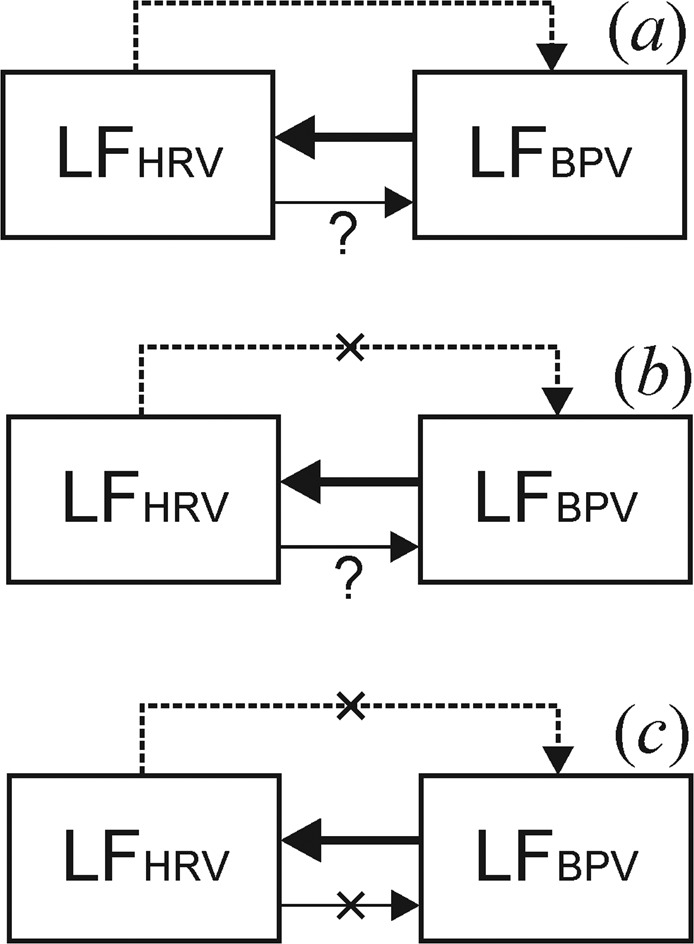
Schematic representation of the structure of couplings between the LF components in the signals of the systems of HR and vascular tone regulation in the normal state (**a**) and during cardiac surgery in CPB without cardioplegia (**b**) and under cardioplegia (**c**). Arrows indicate the directions of couplings. The dashed line is a hemodynamic coupling from HRV to BPV, the solid line indicates an autonomous regulatory influence from BPV to HRV and the thin line indicates an autonomous regulatory influence from HRV to BPV.

We assessed the autonomic regulation of BP using PPGV analysis, taking into account the contribution of the distal arteries to the formation of the PPG finger signal^12^. Under CPB conditions, the hemodynamic coupling between the mechanisms of HRV and BPV autonomic regulation is largely destroyed (the crossed out dotted arrow in Fig. 1) and the neurogenic coupling from BPV to HRV (solid line in Fig. 1) is held. Under conditions of cardioplegia, there is no neurogenic coupling from LF oscillations in HR (as there is no mechanical and electrical function of the heart) to LF oscillations in BP (the crossed out thin line in Fig. 1c). The presence of such coupling in the absence of cardioplegia is an open question.

### Signal processing and data analysis

Before the processing, the studied signals were filtered in the frequency range of 0.04–0.4 Hz (LF + HF band). The spectral analysis of HRV was carried out in accordance with the methodological recommendations^15^. We calculated the following indices of HRV: the total power of HRV spectrum (TP, ms^2^), the ratio of LF components of HRV spectrum to TP measured in percent (LF%), the ratio of HF components of HRV spectrum to TP measured in percent (HF%), and LF/HF ratio.

The PPGV power spectra were estimated directly from the PPG signals. The power spectrum was evaluated by the Welch method^16^ in one-minute windows with a shift of 20 seconds. A triangular spectral window was used for the spectrum estimation. A critical power value was calculated, above which spectral components were considered statistically significant (*p* = 0.05). For this, using the surrogate data, we tested the statistical hypothesis of normal noise bounded in the frequency band 0.04−0.4 Hz. Then we applied the standard HRV analysis technique to the PPGV spectra and calculated the spectral indices LF%, HF%, and LF/HF,

For the PPG data, it is difficult to interpret the absolute values of the waveforms. The output of the PPG sensors is proportional to the unknown coefficient, which depends on optical features of subject’s skin, BP values, sensors placement, optical and electrical characteristics of the sensor, the room illumination and temperature, and other factors. The interpretation of the PPGV absolute values is an open question and goes beyond the scope of the study; instead we used conventional units (cu) to measure the PPG signals. Conventional units are values of the PPG discrete samples and are proportional to the sensor output. Since the proportional coefficient between the blood flow and cu is unknown, it is difficult to interpret the absolute values of LF, HF, and TP. However, LF%, and HF% are dimensionless and have the same interpretation as the respective HRV indexes.

To estimate the synchronization between the LF oscillations in HR and PPG, we applied the method proposed by us recently^14^. Index *S* defines the relative time (in percents) of synchronization between the considered LF oscillations. Each synchronization index *S* was accompanied by an estimate of the probability of randomly obtaining the calculated *S* value for obviously nonsynchronous signals – *p*-value. To evaluate *p*, we used a method for generating surrogate data based on randomizing the phases of the Fourier harmonics of signals^17,18^ and adapted to assess the statistical significance of the results of synchronization detection in the systems under study^13^. In practice, the estimation of index *S* is considered to be statistically significant, if *p* < 0.05.

To analyze the relationship between the spectral components of the LF oscillations in HRV and PPGV, the coherence function *С*(*f*)^19^ and the corresponding critical significance level *p* = 0.05 were calculated. For this purpose, the method of generating surrogate data was used, based on the randomization of the phases of the Fourier harmonics of the signals^17,18^, as well as it was done in the previous method. When the critical significance level for *С*(*f*) was greater than *p* = 0.05 in the corresponding frequency range, the spectral components were considered statistically coherent.

The analysis of directional couplings was carried out using the method of phase dynamics modeling based on a comparison of the prognostic capabilities of individual and joint models describing the dynamics of instantaneous phases of analyzed signals^20,21^. The paper^21^ presents the formulas for estimating from time series the strength *G* of coupling between the systems under study together with a 95% confidence interval at different trial delays *τ*. Coupling is considered to be significant if *G*, together with its confidence interval is above zero. The calculation of characteristics of directional couplings was carried out for the time shifts between the time series from 0 to 20 seconds (up to two characteristic periods of oscillation). The parameter of increase of the instantaneous phase values at each iteration was chosen to be equal to the characteristic oscillation period of 10 seconds in accordance to the paper^22^. Before the processing, the studied signals were filtered in the frequency range of 0.06–0.14 Hz^14^.

Quantitative data are presented below as mean with standard deviation (M ± SD).

## Results

### Cardiopulmonary bypass without cardioplegia

**Figure 2:**
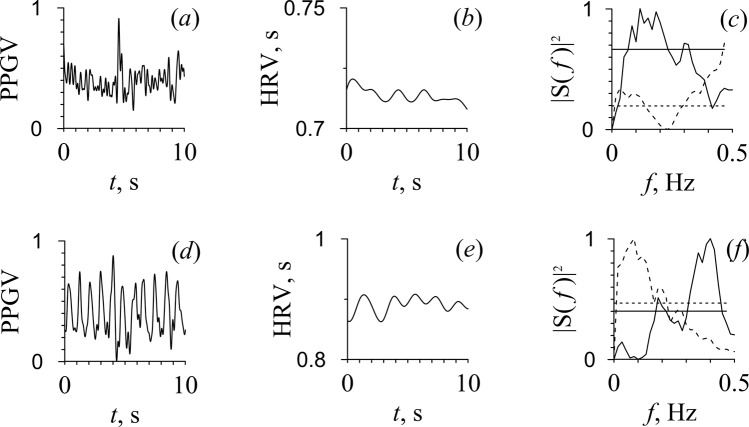
PPG signal (**a**,**d**), RR intervals (**b**,**e**), and their Fourier power spectra (**c**,**f**) depicted by solid line for PPG and by dashed line for RR intervals. The dashed and solid horizontal lines in (**c,f**) correspond to the critical power value (*p* = 0.05), estimated from RR intervals and PPG signals, respectively. The upper line (**a**–**c**) corresponds to patient D with low amplitude of PPG and bottom line (**d**–**f**) corresponds to patient A with a distinguishable pulse wave under CPB.

The low overall HRV level in all five patients and the very low amplitude of PPG (pulse waves were hardly noticeable by visual analysis) in three patients out of five draws attention. Figure 2 shows an example of signals from patient D with low amplitude PPG (Fig. 2a) and from patient A with a distinguishable pulse wave (Fig. 2d). At the same time, a pronounced peak at a frequency of about 0.1 Hz was observed in the spectra of HRV and PPG only in three patients. It should be noted that well-distinguished pulse waves in PPG and a peak at 0.1 Hz in the PPG spectrum were observed in different patients. In the PPGV spectrum, peaks were observed also at the pulsation frequencies generated by the pump oxygenator and mechanical ventilation.

It was also found that in four of five patients, the power of the LF range in HRV and PPGV was less than the power of the HF range (Table 1).

**Table 1:** Spectral components in HRV and PPGV in patients with and without cardioplegia.

Patients	TP_HRV_	LF%_HRV_	HF%_HRV_	LF/HF_HRV_	LF%_PPGV_	HF% _PPGV_	LF/HF_PPGV_	S, %
**Patients without cardioplegia**								
Patient A	0.080	46	39	1.18	11	83	0.13	6
Patient B	0.042	20	73	0.27	49	44	1.11	26
Patient C	0.044	27	63	0.43	15	83	0.18	14
Patient D	0.041	33	54	0.61	36	61	0.59	12
Patient E	0.067	21	66	0.32	27	64	0.42	53
M ± SD	0.054 ± 0.018	30 ± 11	59 ± 13	0.56 ± 0.37	28 ± 15	67 ± 16	0.49 ± 0.40	22.2 ± 18.7
**Patients with cardioplegia**								
Patient F					24	71	0.34	
Patient G					36	57	0.63	
Patient H					27	70	0.38	
Patient I					47	42	1.13	
Patient J					63	23	2.70	
M ± SD					39 ± 16	53 ± 20	1.04 ± 0.98	

M ± SD, mean with standard deviation.

**Figure 3:**
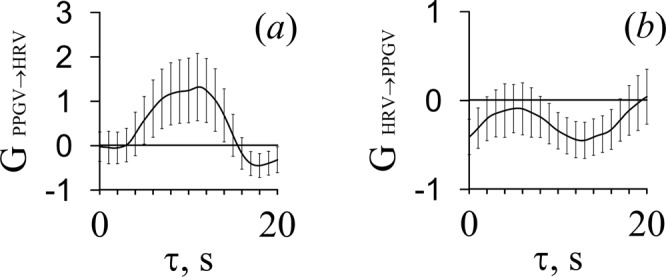
The results of the analysis of directional coupling between the HRV and PPGV signals for patient E at different values of time lags. (**a**) The strength of coupling from PPGV to HRV in the LF range; (**b**) the strength of coupling from HRV to PPGV in the LF range. Vertical lines indicate 95% confidence interval (95% CI).

The analysis of the directionality of couplings through the phase dynamics modeling revealed the presence of coupling from LF oscillations in PPG to LF oscillations in HR for three out of five patients. This result was statistically significant at a 0.05 level for lags from 3.5 to 14.0 seconds (Table 2). An example of phase dynamics analysis is presented in Fig. 3. In particular, in Fig. 3a, the *G*_PPGV→HRV_, together with a confidence interval, lies above zero for a set of values from 4 to 14 seconds. The duration of the interval of 10 seconds corresponds to one characteristic oscillation period, therefore it can be stated that PPGV has an effect on HRV in the LF range with a probability of random error of no more than 0.05. We did not obtain any indication of the presence of coupling from HRV to PPGV.

**Table 2:** Intervals of time lags between the LF oscillations in PPG and HR in patients without cardioplegia.

Patients	^*τ*^GPPGV→HRV > 0^,*S*^
Patient A	—
Patient B	4.0–14.0
Patient C	—
Patient D	3.5–12.0
Patient E	5.0–14.0

For all five subjects, the epochs of synchronization between the LF oscillations in HR and PPG were found out. However, in four patients the values of index *S* were rather small. Only in one case out of five, significant synchronization was revealed (patient E, *S* = 53%, *p* = 0.004) (Table 1).

**Figure 4:**
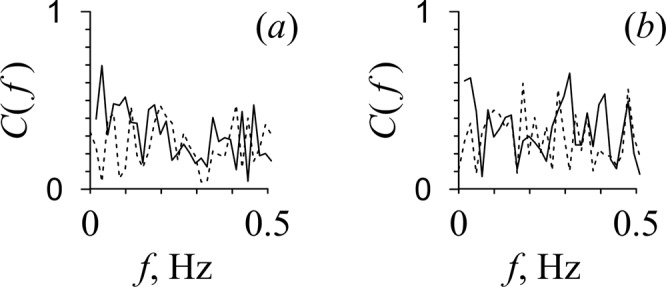
The results of the analysis of the coherence function *C*(*f*) for the HRV and PPGV signals in the LF and HF bands. *C*(*f*) is shown with solid line for patients D (**a**) and E (**b**). The 95% confidence interval is shown with dashed line.

The calculation of the coherence function *C*(*f*) showed that the coherence of the LF oscillations in HRV and PPGV is low and its significance level is close to *p* = 0.05 for four out of five patients. Figure 4a depicts *C*(*f*) for patient D. The values of *C*(*f*) are statistically significant (*p* = 0.05) over the 72% of the LF range. Figure 4b shows *C*(*f*) for patient E, for which the spectral components are statistically insignificant at a frequency of about 0.1 Hz. The maximum statistically significant values (*p* = 0.05) of the coherence function *C*(*f*) for the HRV and PPGV oscillations in the LF range are shown in Table 3.

**Table 3:** Maximum statistically significant values (*p* = 0.05) of the coherence function *C*(*f*) for oscillations in the LF range of HRV and PPGV in patients without cardioplegia.

Patients	*С*(*f*)
Patient A	0.66
Patient B	0.32
Patient C	0.59
Patient D	0.52
Patient E	0.45

### Cardiopulmonary bypass with cardioplegia

**Figure 5:**
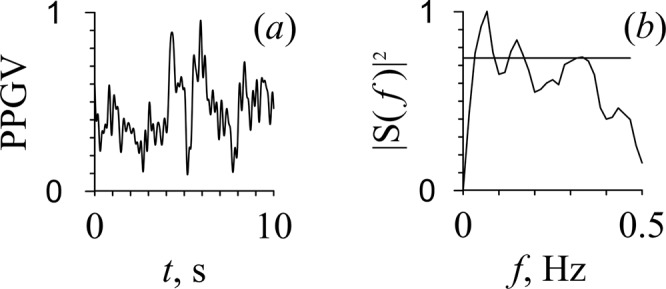
PPG signal (**a**) and its Fourier power spectrum (**b**) for patient G under cardiopulmonary bypass and cardioplegia.

Visually, pulse waves were not noticeable in the PPG signals of all patients (Fig. 5a). Only in three of five subjects the PPGV spectra showed peaks at a frequency of about 0.1 Hz (Fig. 5b). The peaks at the pulsation frequencies produced by the pump oxygenator were not identified in the power spectra. The spectral analysis of PPGV revealed that the power of LF oscillations was less than the power of HF oscillations in three out of five patients (Table 1).

## Discussion

The obtained results agree with a conclusion that coupling from the heart to the peripheral vessels has a hemodynamic nature and the coupling from the vessels to the heart is carried out through the neural pathways^23^. In particular, for three out of five subjects, a significant impact of LF fluctuations in PPG on LF fluctuations in HR was detected. At the same time, in one case, this coupling from the vessels to the heart was so strong that it led to phase synchronization. In this case, there was no significant coherence between the spectral components at a frequency of about 0.1 Hz that eliminates the presence of leakage process (parasitic mixing of signals in the measurement channel), which could lead to a false detection of synchronization.

Detection of weak coupling between signals corrupted with noise is a complex problem. To solve this problem, we used the method based on phase dynamics modeling, which is one of the most sensitive techniques. However, even this method encounters difficulties caused by signal complexity. We revealed the presence of statistically significant coupling from LF oscillations in PPG to LF oscillations in HRV in three out of five patients. To ensure the reliability of the results, we inevitably have to sacrifice the sensitivity of the method. Obtaining a statistically insignificant result of the presence of coupling for the other two patients does not mean its absence, but only indicates that for these signals it is impossible to draw a reliable conclusion about the presence of coupling. For these two patients, the obtained result may be due to a higher noise level, a decrease in coupling strength, or the absence of noticeable oscillations in the studied frequency ranges. A more detailed analysis requires an increase in signal duration or direct registration of sympathetic nerve activity, which is impossible in practice. Therefore, we have to confine ourselves to conclusions based on the analysis of statistically significant results obtained for only a part of patients.

We have not got any results in favor of the hypothesis of the presence of a nervous coupling from the heart to the vessels. Under the hemodynamic dissociation of the heart and vascular bed through the use of CPB, the dynamics of the HRV parameters did not have any effect on the PPGV parameters, despite the continuing possibility of transmitting information from the heart to the vessels along the nerve pathways. We found out that neurogenic coupling from the peripheral vessels to the heart is characterized by small lag times. This may be the basis for the dominance of regulatory coupling from the peripheral vessels to the heart in adaptive processes in the circulatory system. Such coordination interactions are necessary to maintain the functional integrity of the circulatory system.

The presence of LF oscillations in PPG, even if clearly observed in several patients, in the absence of influences from the heart function, confirms the independence of the mechanisms of autonomic regulation of BP through modulation of vascular tone causing their occurrence in this signal. This is consistent with the results of other authors^24^, indicating a lack of changes in BPV in the background of CPB, which is caused by the stability of mechanisms for regulating BP to external influences. At the same time, both the renin-angiotensin and sympathetic systems are involved in the genesis of LF oscillations in BP^24^. According to some authors, the LF component of PPG may be a marker of patient separation according to the state of peripheral vascular resistance^25^.

A number of researchers have shown that the LF oscillations in mean BP are due to changes in peripheral vascular resistance^26,27^. Besides, the low correlation between the LF oscillations in BP and the cardiac state, for example, cardiac output, has been revealed^28^. However, the LF oscillations in cardiac output have shown good correlation with the LF oscillations in HRV^27^. The results of mathematical modeling also confirm the leading role of the tone of peripheral vessels in BP control^29^. Investigations of LF oscillations in BP (so-called Mayer waves) have shown that they are highly coherent with the oscillations in sympathetic nervous activity, baroreflectory control, and liberation of endothelium-derived nitric oxide, which may be important for the spontaneous control of the vessels tone^30^.

Considering data on the significant contribution of BPV to PPGV^1,12^, LF fluctuations in PPG also characterize the baroreflex regulation of BP. We believe that baroreflex regulation and sympathetic effects on vascular tone are components of a single central mechanism for regulating BP, which is involved in the formation of LF oscillations in BP and PPG.

The question of functional autonomy or the dependence of LF fluctuations in HR on the mechanisms of autonomic regulation of BP needs to be clarified. Earlier, we showed the phenomenon of separate capture of LF oscillations in HR and PPG^13^, indicating the autonomy of these oscillations. In the current study, in one patient under cardiopulmonary bypass, significant synchronization of LF oscillations in HR and PPG was also detected. Considering, as mentioned above, the lack of coupling in the direction from the heart to the vessels, but the presence of LF oscillations in HR, synchronization could be actively imposed by the processes of autonomic regulation of peripheral vascular resistance through the vasomotor centre. The lack of significant synchronization in the other patients under conditions of cardiac surgery with extracorporeal circulation is probably due to severe autonomic dysfunction against the background of chronic cardiovascular pathology and acute operational stress, which caused reduced activity of LF oscillations during the registration of physiological signals.

It should be noted that regarding the nature of LF oscillations in PPG, besides the hypothesis described above in the text, there are other opinions.

A number of authors consider the PPG oscillations in the LF range to be a reflection of vasomotions (vascular endothelial activity, myogenic autoregulation of blood flow, and regulation of skin temperature)^31,32^ and passive fluctuations in peripheral blood flow, which are conditioned by the response of elastic arteries and veins to the oscillations in BP, HR, and systolic blood flow^33^. Ferrario *et al*.^34^ showed the key role of central volume in the origin of LF oscillations of HR. Perhaps this result can be attributed to BPV. The complex nature of LF oscillations in PPG (for example, this issue was discussed in detail by Baselli *et al*.^35^) may involve all of the above-mentioned processes to some extent.

The lack of characteristic peaks in the LF range of the HRV and PPGV spectrum in some patients in the presence of significant coupling and synchronization may be due to difficulties in visualizing LF oscillations using spectral analysis methods because of the aforementioned marked reduction in their power. Also important is the fact of detecting a peak at 0.1 Hz in the spectrum of PPG even in patients with visually poorly noticeable pulse wave at PPG (including the case of cardioplegia), indicating the presence of these fluctuations in vascular tone under conditions of low blood filling.

Taking into account the features of the study (mechanical ventilation, CPB, cardioplegia, and cardiac surgery on the heart) we should carefully interpret the results of spectral analysis of HRV and PPGV, since there are no other studies with which to compare the results. For example, it is known that anesthesia can weaken the Mayer waves and decrease the spontaneous baroreceptor modulation^36^. However, such observations were not previously performed directly during cardiac surgery. Therefore, the obtained results of spectral analysis should be carefully compared with the results obtained for qualitatively different categories of subjects. The interpretation of the results should be carried out in conjunction with other methods of analysis.

We analyzed the data obtained under rather difficult experimental conditions. Therefore, the analyzed experimental ensemble is small. However, we investigated the qualitative effects. We believe that detecting these effects even in a few particular recordings is beneficial and forwards the understanding of cardiovascular system and its regulation. The conclusions we made are verified by careful statistical analysis based on the ensemble of surrogate signals that reproduce the statistical (spectral) characteristics of the experimental data. The surrogate data themselves can be considered as an extension of experimental data. This justifies the reliability of the obtained results and conclusions on a small experimental ensemble. However, the great variability in the characteristics of the cardiovascular system makes it desirable to expand the experimental ensemble. With a larger set of signals, it may be possible to uncover other effects that do not appear in analyzed recordings.

## Conclusion

The mechanisms of autonomic regulation of peripheral vascular resistance, the activity of which is manifested in LF fluctuations in PPG, are autonomous from the mechanisms of autonomic regulation of the heart. Moreover, these mechanisms of regulation of the heart and blood vessels to some extent retain their activity under conditions of CPB and cardioplegia (the latter refers only to LF fluctuations in PPG). The coordinating coupling from the vessels to the heart is carried out by a neurogenic route, whereas the coupling in the opposite direction has a hemodynamic nature (due to cardiac output).
